# Dendritic cell-derived nitric oxide inhibits the differentiation of effector dendritic cells

**DOI:** 10.18632/oncotarget.11361

**Published:** 2016-08-18

**Authors:** Chuanping Si, Ruihua Zhang, Tianshu Wu, Geming Lu, Yuan Hu, Hui Zhang, Feihong Xu, Peter Wei, Kang Chen, Hua Tang, Garabet Yeretssian, Huabao Xiong

**Affiliations:** ^1^ Institute of Immunology and Molecular Medicine, Jining Medical College, Shandong 272067, China; ^2^ Department of Medicine, Immunology Institute, Icahn School of Medicine at Mount Sinai, New York, NY 10029, USA; ^3^ Department of Obstetrics and Gynecology, Wayne State University, Detroit, MI 48201, USA; ^4^ Institute of Immunology, Taishan Medical University, Tai'an, Shandong 271000, China

**Keywords:** dendritic cell, iNOS

## Abstract

Dendritic cells (DCs) play a pivotal role in the development of effective immune defense while avoiding detrimental inflammation and autoimmunity by regulating the balance of adaptive immunity and immune tolerance. However, the mechanisms that govern the effector and regulatory functions of DCs are incompletely understood. Here, we show that DC-derived nitric oxide (NO) controls the balance of effector and regulatory DC differentiation. Mice deficient in the NO-producing enzyme inducible nitric oxide synthase (iNOS) harbored increased effector DCs that produced interleukin-12, tumor necrosis factor (TNF) and IL-6 but normal numbers of regulatory DCs that expressed IL-10 and programmed cell death-1 (PD-1). Furthermore, an iNOS-specific inhibitor selectively enhanced effector DC differentiation, mimicking the effect of iNOS deficiency in mice. Conversely, an NO donor significantly suppressed effector DC development. Furthermore, *iNOS*^−/−^ DCs supported enhanced T cell activation and proliferation. Finally iNOS^−/−^ mice infected with the enteric pathogen *Citrobacter rodentium* suffered more severe intestinal inflammation with concomitant expansion of effector DCs in colon and spleen. Collectively, our results demonstrate that DC-derived iNOS restrains effector DC development, and offer the basis of therapeutic targeting of iNOS in DCs to treat autoimmune and inflammatory diseases.

## INTRODUCTION

The delicate immunological equilibrium between inflammation and tolerance is essential for the host to mount effective and controlled immune defense against noxious pathogens without causing destructive immunity against innocuous self-tissues or commensal microbes [1, 2]. Recent findings suggest that cells of both the innate and adaptive immune systems are important players in the decision between effector immunity and immune tolerance. Among the various immune cells, dendritic cells (DCs) are critical in processing and integrating various immunological cues to instruct the development of adaptive immunity. For example, effector DCs that secrete interleukin-12 (IL-12), tumor necrosis factor (TNF), IL-6 and interferon-γ (IFN-γ) can promote T helper (Th) cell differentiation into Th1, Th2 or Th17 effector subsets respectively, thereby tailoring adaptive immunity to effectively control and eradicate infections. Regulatory DCs that express IL-10, programmed cell death-1 (PD-1), transforming growth factor-β (TGF-β) and argininase suppress effector T cell activation and promote regulatory T cell (Treg) differentiation to enforce immune tolerance to self and commensal antigens. Therefore, uncontrolled effector or regulatory functions of DCs can result in either autoimmune and inflammatory diseases or pathological immune suppression. Despite the knowledge of many DC-extrinsic signals that influence effector and regulatory DC differentiation, DC-intrinsic mechanisms that underlie the differentiation of effector and regulatory DC cells are poorly understood.

Inducible nitric oxide synthase (iNOS) activity has been demonstrated in a wide array of cells and tissues [3, 4]. Nitric oxide (NO), the enzymatic product of iNOS, has diverse and important roles in host defense against a variety of pathogens [5, 6], neurotransmission, vascular functions, and immune regulation [8–12, 23]. NO is one of the smallest known bioactive products of mammalian cells. It is clear that NO is an important pro-inflammatory cytotoxic mediator that defends the host against various pathogens by inactivating and destroying infectious agents [13]. Interestingly, NO also plays critical roles in immune suppression [14]. Increasing evidence indicates that NO can affect the function of diverse immune cells, such as macrophage, T cells and DCs [8, 11, 13, 25]. However, it is not clear whether all immune cells express iNOS and what the function and mechanism of immune cell-derived iNOS is, particularly in the regulation of immunity and tolerance in autoimmune and inflammatory diseases [9, 10]. In this study, we show that DC-intrinsic iNOS is required to suppress effector DC differentiation and limit pathogenic inflammatory CD4^+^ T cell responses. DC-derived NO suppresses NF-kB signaling and inflammasome activation. Our results suggest that targeting DC-intrinsic iNOS may represent an efficient therapeutic strategy for immune and inflammatory diseases.

## RESULTS

### iNOS-deficient DCs exhibit enhanced effector differentiation

**Figure 1:**
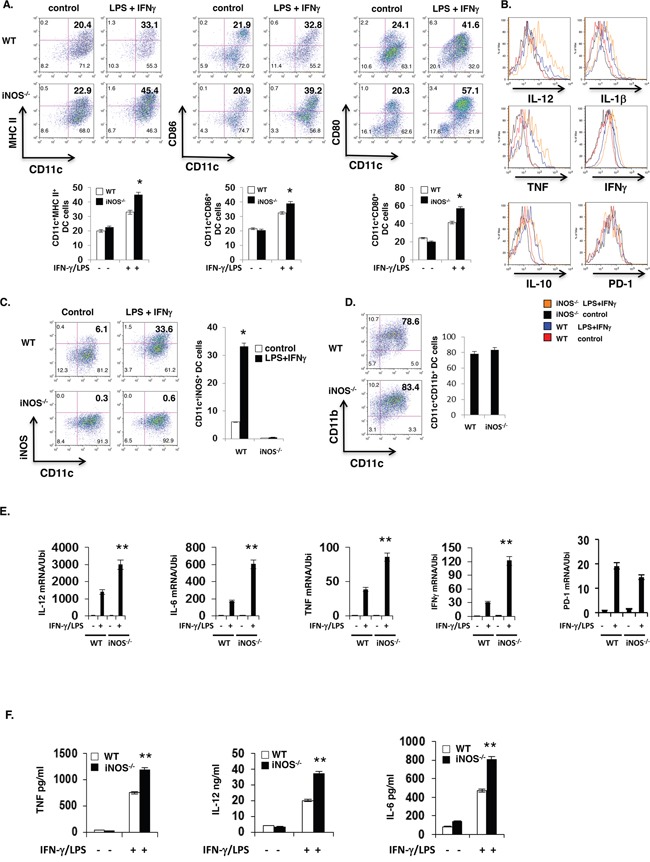
More maturation and Enhanced effector DC differentiation in iNOS-deficient mice **A.** Bone marrow cells from wild type or iNOS^−/−^ mice were cultured with GM-CSF (10ng/ml) and IL-4 (10ng/ml) for 7 days, then stimulated with IFN-γ (10ng/ml) plus LPS (100ng/ml) for 24 h, maturation markers including MHC II, CD80 and CD86 expression in CD11b^+^CD11c^+^ cells were analyzed by FACS. **B.** Cells prepared in (A) were intracellular and surface stained for molecules of effector and regulatory DC in CD11b^+^CD11c^+^ cells by FACS. **C.** The cells prepared in (A) and iNOS expression in CD11b^+^CD11c^+^ cells was determined by FACS. **D.** The purity of CD11b^+^CD11c^+^ cells population in (A) were analyzed by FACS for cell surface staining. **E.** The cells prepared in (A) and mRNA expression of indicated genes was determined by qPCR. **F.** The supernatants in (A) were analyzed by ELISA. Data represent mean ± SD. * P<0.05. **P<0.01.

To investigate the function of NO in effector and regulatory DC differentiation, we first assessed the impact of iNOS deficiency on DC maturation ex vivo. Based on bone marrow-derived DCs (BMDCs) culture, we obtained the 85% to 90% purity of BMDCs in WT or iNOS deficiency mice (Figure 1D and Supplementary Figure S1A). Lipopolysaccharide and IFN-γ treatment caused enhanced maturation of granulocyte and macrophage colony stimulating factor (GM-CSF)- and IL-4-differentiated *iNOS*^−/−^ CD11b^+^CD11c^+^ BMDCs than WT BMDCs, as evidenced by high expression of major histocompatibility complex class II (MHC-II), CD86 and CD80 (Figure 1A). *iNOS*^−/−^ BMDCs also showed higher expression of the effector DC markers TNF, IL-12/IL-23p40 and IL-1β but not the regulatory DC markers IL-10 and PD-1 than WT BMDCs after LPS and IFN-γ stimulation (Figure 1B and Supplementary Figure S2). Consistently, quantitative real-time PCR analysis detected increased induction of IL-12, IL-6, TNF and IFN-γ transcripts in LPS and IL-4-stimulated *iNOS*^−/−^ BMDCs (Figure 1E), and ELISA found that *iNOS*^−/−^ BMDCs secreted more TNF, IL-12 and IL-6 after stimulation (Figure 1F). Our result showed that iNOS expression on BMDCs of WT mice with LPS/IFN-γ stimulation was significantly increased by intracellular staining of CD11b+CD11c+ BMDCs (Figure 1C). Of note, the enhanced effector differentiation of *iNOS*^−/−^ BMDCs was not a result of reduced IL-10 expression, as WT and *iNOS*^−/−^ BMDCs had similar IL-10 after stimulation (Figure 1B). We found that iNOS deficient mice showed normal development in T cells population and DCs percentages compared with that in WT mice (Supplementary Figure S1B) and plasmacytoid dendritic cells (pDC) maturation was also affected treatment with NO in 7 days pDC culture *in vitro* (Supplementary Figure S3 and Supplementary Figure S4). Taken together, these results show that DC-intrinsic iNOS function inhibits effector DC maturation and differentiation.

### NO-extrinsic inhibits effector DC differentiation

**Figure 2:**
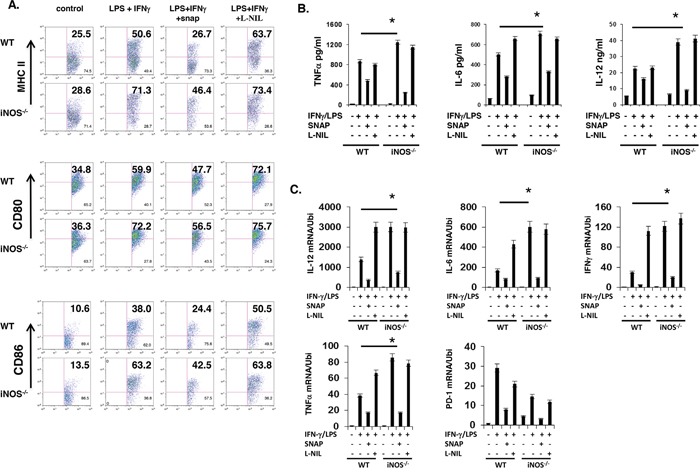
NO suppresses maturation and differentiation of effector DC *in vitro* **A.** Bone marrow cells from WT and iNOS^−/−^ mice were cultured with GM-CSF (10ng/ml) and IL-4 (10ng/ml) for 7 days, then stimulated with IFN-γ (10ng/ml) plus LPS (100ng/ml) for 24 h in the presence of SNAP or L-NIL, DC cell maturation markers and effector or regulatory DC cells markers in CD11b^+^CD11c^+^ were analyzed by FACS. **B.** The supernatants prepared in (A) were analyzed by ELISA as indicated in Figure. **C.** The cells prepared in (A), total RNA was extracted, and qPCR was performed for the analysis of effector or regulatory DC molecules as indicated in Figure. Data represent mean ± SD. * P<0.05. **P<0.01.

To directly determine if NO-extrinsic inhibits effector DC differentiation, we stimulated BMDCs *in vitro* with IFN-γ and LPS for 24h in the absence or presence of the iNOS-independent NO donor S-Nitroso-N-acetylpenicillamine (SNAP) or the iNOS inhibitor L-N6-(1-Iminoethyl)lysine (L-NIL), and examined DC maturation and effector molecule expression by flow cytometry. IFN-γ and LPS induced higher proportions of MHC-II^+^, CD80^+^ and CD86^+^ cells in *iNOS*^−/−^ BMDCs than WT BMDCs (Figure 2A). Consistent with an inhibitory role of NO in effector DC differentiation, SNAP significantly reduced IFN-γ- and LPS-induced up-regulation of MHC-II, CD80 and CD86 in both WT and *iNOS*^−/−^ BMDCs, whereas L-NIL enhanced the up-regulation of these markers in WT but not in *iNOS*^−/−^ BMDCs (Figure 2A). Both ELISA and quantitative real-time PCR (QRT-PCR) confirmed that SNAP suppressed the expression of TNF, IL-6 and IL-12 by WT and *iNOS*^−/−^ BMDCs following IFN-γ- and LPS stimulation, while L-NIL enhanced the induction of the production of these effector cytokines only in WT BMDCs (Figure 2B and 2C). Of note, taken together, these results suggest that NO-extrinsic suppresses the differentiation of effector DCs but it has no effects on regulatory DCs.

### *iNOS*^−/−^ effector DCs induce enhanced CD4^+^ T cell response

**Figure 3:**
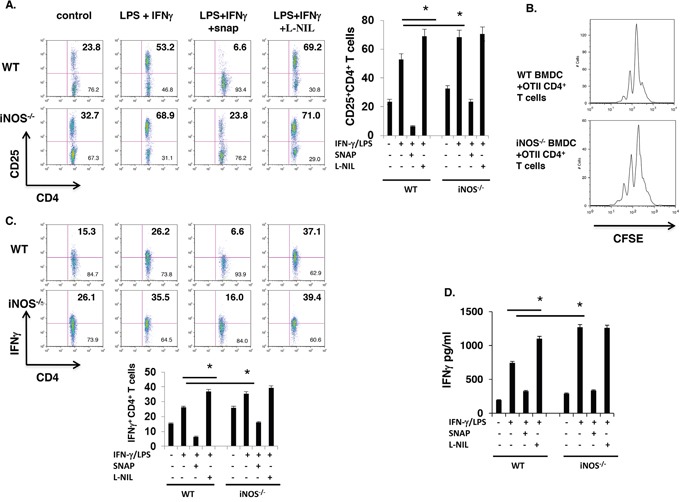
iNOS^−/−^ effector DCs induce enhanced CD4+ T cell activation **A.** Bone marrow cells from WT and iNOS^−/−^ mice were cultured with GM-CSF (10ng/ml) and IL-4 (10ng/ml) for 7 days, then stimulated with IFN-γ (10ng/ml) plus LPS (100ng/ml) for 24 h, then BMDCs were thoroughly washed with medium and were irradiated with 2000 rad, CD4^+^ T cells purified from spleen and lymph nodes of OTII transgenic mice were co-incubated with these WT or iNOS^−/−^ BMDCs for 3 days in present of OTII peptide. CD25 expression on T cells as activated markers were stained by FACS. **B.** BMDCs were prepared as in (A) and CD4^+^ T cells purified from spleen and lymph nodes of OTII transgenic mice and then were labelled with CFSE as indicating T cells proliferation status, CFSE labelled T cells were co-incubated with irradiated WT or iNOS^−/−^ BMDCs for 3 days in present of OTII peptide, proliferation of CD4^+^ T cells was analyzed by FACS. **C.** BMDCs and CD4+ T cells were prepared in (A) and were co-incubated with irradiated WT or iNOS^−/−^ BMDCs for 3 days in present of OTII peptide, then stained for intracellular IFN-γ in CD4^+^ T cells by flow cytometry. **D.** IFN-γ production in the supernatants prepared in (A) was analyzed by ELISA. Data represent mean ± SD. * P<0.05. **P<0.01.

Above results showed that DC-derived iNOS and iNOS released NO or NO-extrinsic affected the maturation and differentiation of effector DCs in BMDCs *in vitro* culture, and to determine whether these enhanced maturation of effector DCs from iNOS deficiency mice could induce more higher T cell activation and response, we obtained bone marrow cells from iNOS^−/−^ or WT control mice and were incubated with GM-CSF (10 ng/ml) plus IL-4 (10 ng/ml) *in vitro* for 7 days. The cells were then activated with LPS (100 ng/ml) plus IFN-γ (10 ng/ml) for overnight. After confirmation of effector DCs maturation markers including MHCII-, CD80- and CD86-positive cells and differentiation markers including TNF−, IL-6- and IL-12/IL23p40- in CD11b^+^CD11c^+^ double positive BMDCs, we co-cultured WT or iNOS^−/−^ DCs with OTII CD4^+^ T cells. CFSE dilution assay indicated that T cell proliferation was significantly enhanced in cultures with iNOS^−/−^ DCs than that with WT DCs (Figure 3B), suggesting that iNOS deficiency in DCs induce more T cell proliferation, and the activation markers including CD25 was significantly increased in CD4^+^ T cells co-cultured with iNOS-deficient DCs (Figure 3A). Furthermore, the population of IFN-γ-producing T cells and production of IFN-γ was significantly enhanced in cultures with iNOS deficient DCs (Figure 3C and 3D). Taken together, the results suggest that iNOS^−/−^ effector DCs induce stronger T cell activation and response.

### DC-intrinsic iNOS regulates effector DC differentiation *in vivo*

**Figure 4:**
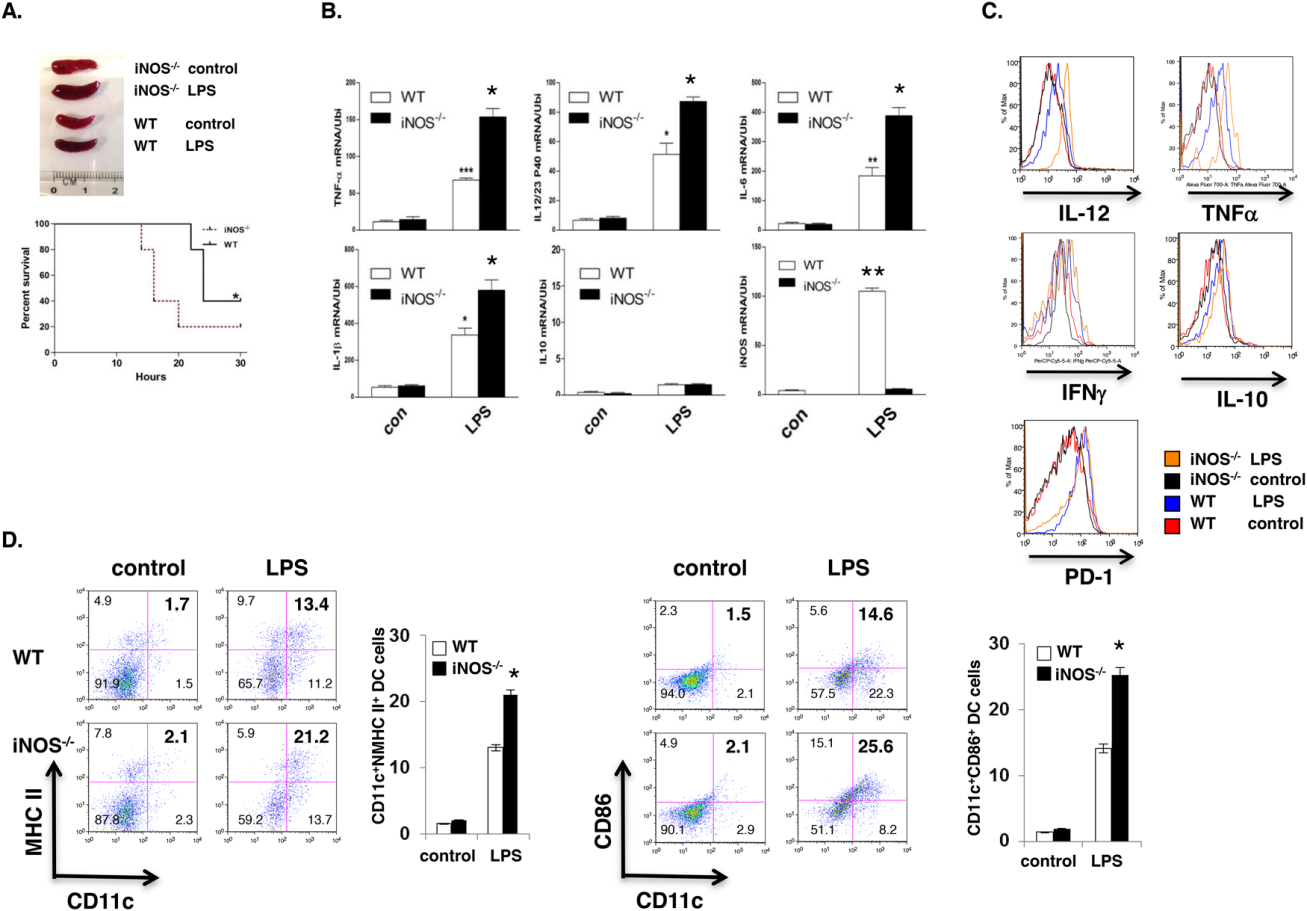
iNOS deficiency promotes the effector DC differentiation *in vivo* **A.** WT or iNOS^−/−^ mice were injected (i.p) with LPS for 24 hours. Spleen morphology and mice survival rate was observed. **B.** Total RNA from spleen of WT or iNOS^−/−^ mice after LPS injection as in (A) was extracted for qPCR. **C.** Effector/stimulatory DC marker in CD11b^+^CD11c^+^ prepared from spleens of WT or iNOS^−/−^ mice after LPS injection as in (A) and analyzed by FACS. **D.** Maturation markers in CD11b^+^CD11c^+^ cells of mesenteric lymph node of WT or iNOS^−/−^ mice after LPS injection as in (A) were analyzed by FACS. Data represent mean ± SD. * P<0.05. **P<0.01.

**Figure 5:**
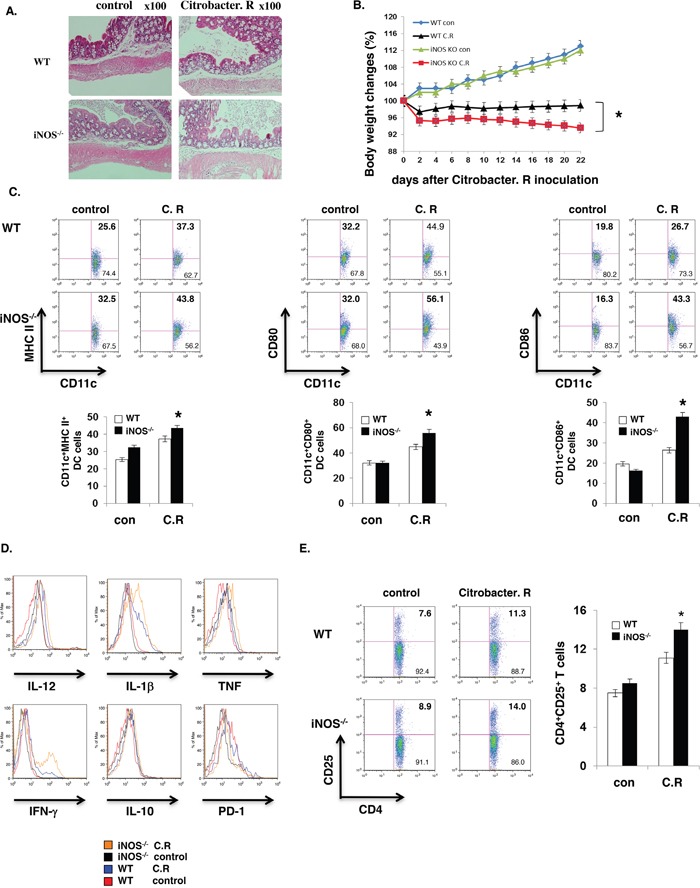
iNOS suppress effector DC differentiation *in vivo* WT or iNOS^−/−^ mice were fed orally with Citrobacter Rodentium. Colon tissues with H&E staining were showed as **A, B.** Body weights of WT or iNOS^−/−^ mice which were fed orally with Citrobacter Rodentium were balanced every two days. **C.** Maturation markers in CD11b^+^CD11c^+^ cells of mesenteric lymph node of WT or iNOS^−/−^ mice which were fed orally with Citrobacter Rodentium as in (A) and were analyzed by FACS. **D.** Effector/stimulatory DC markers in CD11b^+^CD11c^+^ cells of mesenteric lymph node of WT or iNOS^−/−^ mice which were fed orally with Citrobacter Rodentium as in (A) and were analyzed by FACS. **E.** T cell activation marker CD25 expression in CD4^+^ T cells of mesenteric lymph node of WT or iNOS^−/−^ mice which were fed orally with Citrobacter Rodentium as in (A) were analyzed by FACS. Data represent mean ± SD. * P<0.05. **P<0.01.

It is well-established that iNOS-derived NO plays an important role in host defense against bacterial infection by killing bacteria directly. Accumulating evidence indicates that iNOS-deficient mice are susceptible to bacterial infection. However, an important question remains unaddressed yet, what is the state of dendritic cells differentiation in iNOS-deficient mice during bacterial infection? To answer this question, first, we injected LPS (800mg/mouse) by intraperitoneally into WT and iNOS^−/−^ mice for 8 hours and mice were then sacrificed or measured the survival rate. LPS induced more severe endotoxin shock in iNOS-deficient mice with much enlarged spleen size and significantly higher lethality compared with WT mice (Figure 4A), and these phenomena indicated that LPS induced more severe response in iNOS-deficient mice. Interestingly, the production of effector DCs signature genes including TNF and IL-12/IL-23p40, IL-6, and IL-1β in spleen was clearly enhanced in *iNOS*^−/−^ mice, but the IL-10 gene expression in iNOS^−/−^ mice was comparable with that in WT mice after LPS injection (Figure 4B). In addition, maturation and differentiation signatures of CD11b^+^CD11c^+^ effector DCs in spleen including MHC II, CD86 and IL-12/IL-23p40, TNFα, IFN-γ were also increased in *iNOS*^−/−^ mice after LPS injection, the regulatory DC markers including IL-10 and PD-1 were not different between WT and iNOS^−/−^ mice (Figure 4D and Figure 4C). Again we injected by oral inoculation into WT and iNOS^−/−^ mice with *Citrobacter Rodentium* (2 × 10^9^ CFU per mouse) for 3 weeks and mice were then sacrificed. Bacterial induced colitis in iNOS-deficient mice were significantly severe compared with WT mice (Figure 5A and 5B). Interestingly, both maturation and differentiation signatures of CD11b^+^CD11c^+^ effector DCs in spleen including MHC II, CD80, CD86 and IL-12/IL-23p40, TNF, IFN-γ, IL-1β were obviously increased in *iNOS*^−/−^ mice after *Citrobacter Rodentium* infection, but these signatures in regulatory DCs including IL-10 and PD-1 were not enhanced in iNOS^−/−^ mice (Figure 5C and Figure 5D). More T cell proliferation marker of CD25 expression in CD4+ T cells was induced in mesenteric lymph nodes of iNOS deficiency mice which Citrobacter Redentium induced colitis than that in WT control mice (Figure 5E). These results indicate that effector DCs differentiation and colitis was actually enhanced in *iNOS*^−/−^ mice following the bacterial infection. Thus, iNOS deficiency in DCs promotes effector DCs differentiation in infection models, suggesting that iNOS expressed in DCs may play a negative role in the regulation of effector DC differentiation in innate and adoptive immune response.

### DC-intrinsic iNOS controls DCs function by suppressing NF-κB and inflammasome activity

**Figure 6:**
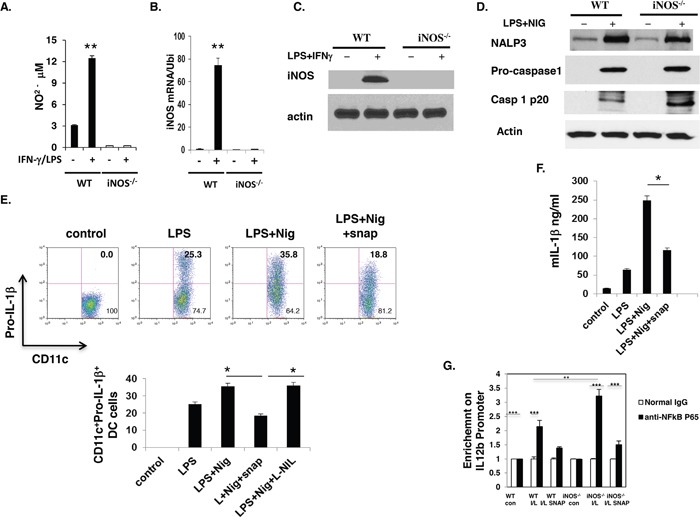
iNOS expression was induced in WT dendritic cells and NO suppress inflammasome activity Bone marrow cells from WT and iNOS^−/−^ mice were cultured with GM-CSF (10ng/ml) and IL-4 (10ng/ml) for 7 days, then stimulated with IFN-γ (10ng/ml) plus LPS (100ng/ml) for 24 h. **A.** NO release in culture supernatant were analyzed. And **B.** total RNA was prepared in (A) and iNOS mRNA expression was analyzed by qPCR. **C.** The cell lysates prepared in (A) were prepared and western blotting was performed for the analysis of iNOS protein expression in these cells. **D.** Bone Marrow cells from wild type mice were cultured with GM-CSF and IL-4 for 7 days, and then stimulated with LPS for 5 h before stimulation with 20μM Nigericin for 30 min. To evaluate the effect of NO, the indicated concentration of SNAP or L-NIL were added to the cell culture 30 min prior to stimulation with nigericin. The cells lysate were prepared and western blotting was performed for the evaluation of inflammasome activity in BMDC cells. **E.** The cells prepared in (D) Pro-IL-1β expression in CD11b^+^CD11c^+^ cells was analyzed by FACS. And **F.** IL-1β in culture supernatant was analyzed. **G.** BMDCs from WT and iNOS^−/−^ mice were stimulated with IFN-γ (10 ng/ml) and LPS (200 ng/ml) in the presence of SNAP (500 μM) or L-NIL (40 μM) overnight, followed by ChIP assay. Three micrograms of an anti-NF-kB p65 antibody or isotype-matched IgG as control antibody were used in the immunoprecipitation step. PCR was used to quantify the amount of precipitated DNA with primers flanking the p65-binding site of the IL-12 promoter region. Each bar represents mean ± S.D. from three independent experiments, unpaired Student's t-test, *P<0.05, versus WT cells.

Previous reports showed that iNOS expressed in immune cells and played an important role for Th17 cell differentiation and M1 macrophage differentiation, iNOS expression is modulated following the activation of the immune cell [21, 22]. We hypothesize that NO derived from DCs regulates the function of DCs and to explain the molecular mechanism of DCs-derived iNOS affect DCs function. First we investigate whether DCs express iNOS and release NO after activation. Bone marrow-derived dendritic cells (BMDC) from WT and iNOS^−/−^ mice were activated by LPS plus IFN-γ for 24 h. iNOS expression in CD11b^+^CD11c^+^ cells and NO release in culture supernatant were obviously induced in WT mice (Figure 1C and Figure 6A-6C). Western blotting showed that iNOS protein was indeed induced in WT BMDCs but not in iNOS^−/−^ cells (Figure 6C). iNOS mRNA expression was induced in WT BMDCs (Figure 6B). Based on these preliminary data and other group's reports [21, 22, 10, 11], we evaluate how NO affect the NF-κB pathway and inflammasome activity which were involved in the relative proinflammatory cytokines production (Figure 6D-6G). Previously data, Xiong et al showed that NO significantly suppresses interleukin-12 p40 transcription and NF-κB activation in murine macrophages and dendritic cells [15]. K Mao et al identified NO as a critical negative regulator of the NLRP3 inflammasome via the stabilization of mitochondria, and they showed that nitric oxide (NO) inhibited the NLRP3-mediated ASC pyroptosome formation, caspase-1 activation and IL-1β secretion in myeloid cells from both mice and humans [31]. In order to confirmation NO suppress the inflammasome activity, we culture the BMDCs and induce inflammasome activity by Nigericin, really we found that one of the important indicators of inflammasome activity: caspase 1 p20 protein expression was significantly increased in iNOS deficient dendritic cells by western blot (Figure 6D). And another indicator of inflammasome activity markers: pro-IL-1β expression in BMDCs was affected by NO-extrinsic (Figure 6E and Figure 6F). In addition, to determine whether NO suppress NF-kB signaling pathway, BMDCs from WT and iNOS^−/−^ mice were stimulated with IFN-γ (10 ng/ml) and LPS (200 ng/ml) in the presence of SNAP (500 μM) or L-NIL (40 μM) overnight, followed by ChIP assay. The anti-NF-kB p65 antibody or isotype-matched IgG as control antibody were used in the immunoprecipitation step. PCR was used to quantify the amount of precipitated DNA with primers flanking the p65-binding site of the IL-12 promoter region. the data demonstrated that the DNA binding of NF-kB to the promoter region of IL-12 gene was significantly enhanced in iNOS deficient dendritic cells (DC) compared with WT cells (Figure 6G). Taken together, Nitric oxide release from DC-derived iNOS regulates DCs function by suppression of NF-kB pathway and inflammasome activity.

## DISCUSSION

Effector DCs secrete cytokines, such as IL-12, TNF, IL-6 and IFN-γ, to induce adaptive immune response, including enhancing T cell differentiation toward Th1, Th2, or Th17 subsets, which in turn promotes pathogen clearance and can also result in tissue damage. Regulatory DCs, in contrast, contribute to immune tolerance by triggering T cell apoptosis or anergy or inducing Treg differentiation via the production of regulatory cytokines such as IL-10. Our results showed that *iNOS*^−/−^ mice displayed enhanced effector DC maturation and differentiation following LPS and IFN-γ stimulation. Consistently, molecules associated with effector DCs, including IL-12/IL-23p40, IL-1β and TNF, but not those associated with regulatory DCs, such as IL-10 and PD-1, were elevated in iNOS^−/−^ BMDCs. Therefore, iNOS-mediated NO production by DCs specifically inhibits the differentiation of effector DCs. Three distinct isoforms of NO synthase (NOS) have been identified: neuronal NOS (nNOS), inducible NOS (iNOS), and endothelial NOS (eNOS). These enzymes are products of different genes, with different regulation, localizations, and catalytic properties. nNOS and eNOS are primarily expressed in neurons and endothelial cells, and they are calcium dependent. Recently the data showed that nNOS and eNOS are existing in other cells. iNOS can be induced by cytokines and other stimuli in many cell types. iNOS activity has been demonstrated in a wide array of cells and tissues [3, 4]. In contrast, nNOS and eNOS exist in the cell as preformed proteins whose activity is switched on by the elevation of intracellular Ca2+ concentrations and the binding of calmodulin in response to neurotransmitters or vasoactive substances. Beyond this basic paradigm, additional levels of regulation exist for all three NOS isoforms that may operate during immune responses. Christian Bogdan reported that the expression and function of NOS isoforms in phagocytes, dendritic cells, NK cells and T and B cell lines following the cytokines or other stimuli were mainly iNOS [34]. Its enzymatic product, NO, is one of the smallest known bioactive products of mammalian cells that has diverse functions in neurotransmission, vascular functions, host defense and immune regulation [8–12, 23]. In immune cells, iNOS can be induced by multiple stimuli, including LPS, IFN-γ and TNF, in a calcium-independent manner [3]. NO can function as a pro-inflammatory cytotoxic molecule that mediates host defense by inactivating and destroying infectious agents [13]. Interestingly, NO also plays critical roles in immune suppression [14]. Despite the growing evidence showing that NO may affect responses of a myriad of immune cells, including macrophages, T cells and DCs [8, 11, 13, 25], the function of immune cell-derived NO is unclear, particularly its role in the regulation of immunity and tolerance [9, 10]. We recently demonstrated that T cell-derived iNOS negatively regulates Th17 cell differentiation by nitration of the transcription factor retinoid-related orphan receptor γ t (RORγt) [15]. This current study, together with our earlier finding showing the enhancement of polarization of M1 but not M2 macrophages in iNOS deficiency [22], suggest that NO controls T cell-mediated immunity at multiple stages of effector T cell differentiation.

iNOS-deficient mice displayed an increased population of effector DC phenotype including IL-12, TNF and IL-6 *in vivo and in vitro*. In addition, iNOS deficiency resulted in higher expression of MHC-II and co-stimulatory molecules CD80 and CD86 in mouse BMDCs following LPS and IFN-γ stimulation, thereby endowing these DCs with superior capability of presenting antigens and activating CD4^+^ T cells. Mechanistically, the inhibitory effect of NO in effector DC differentiation may be contributed by its suppressive activity on NF-κB signaling and inflammasome activation. *iNOS*^−/−^ mice orally infected with *C. Rodentium* developed more severe colitis than WT mice and harbored a markedly expanded population of effector DCs in the spleen and colon [30]. The results indicated that DC-intrinsic iNOS and DC-derived NO correlate with aggravated pathogenic inflammatory response. Similar results were observed in the endotoxin-induced sepsis model, where DC-derived iNOS negatively controls effector DC development. Hence, our study offers the basis of targeting DC-intrinsic iNOS as a therapeutic strategy to combat autoimmune and inflammatory diseases.

## MATERIALS AND METHODS

### Mice

C57BL/6J (B6, stock#000664), iNOS^−/−^ mice (B6.129P2-Nos2tm1Lau/J, stock#002609) and CD4^+^ OVA TCR-transgenic (OT-II) mice (B6. Cg-Tg (TcraTcrb)425Cbn/J, stock#004194) were obtained from Jackson laboratory and maintained in the barrier facility at the Mount Sinai School of Medicine. For all the experiments, 6- to 8-week-old female mice were used. The animal study protocols were approved by the Institutional Animal Care and Use Committees of Mount Sinai School of Medicine and Virginia Tech.

### Antibodies

The following antibodies were applied: inos2 from Santa cruz, (USA); actin antibody was obtained from sigma (USA). For flow cytometry, fluorescently labelled antibodies to CD11b (M1/70, FITC-labelled), MHC II (M5/114.15.2, alexa flour 700-labelled), NOS2 (CXNFT, APC-labelled), IFN-γ (XMG1.2, APC-labelled, CD4 (GK1.5, FITC-labelled) were all from eBioscience (USA), and were used at a 1:100 dilution. Antibodies for IL12 P40 (C15.6, PE-labelled) and CD25 (7D4, PE-labelled) were from BD Bioscience at a dilution of 1:100. Antibody for IL-1β (JAMA-147, APC-labelled) from Biolegend was diluted at 1:100.

### Activation of NLRP3 inflammasome

mouse BMDCs were primed with 1μg/mL LPS for 5 h before stimulation with 20μM Nigericin for 30 min. To evaluate the effect of NO, the indicated concentration of SNAP or L-NIL were added to the cell culture 30 min prior to stimulation with nigericin.

### Preparation of bone marrow-derived dendritic cells

Bone marrow (BM) cells were isolated from tibias and femurs of C57BL/6 mice or *iNOS*^−/−^ mice, and Cells were then incubated at 37°C for 3 h and adherent cells were removed. The non-adherent cells were cultured in complete RPMI1640 medium supplemented with 10ng/ml GM-CSF and 10ng/ml IL-4. On day 7, cells were harvested and seeded in fresh complete RPMI1640 medium at a density of 2×10^6^ cells/ml for subsequent experiments.

### Intracellular staining and flow cytometry

Bone marrow-derived macrophages were either activated with 200ng/ml LPS and 10ng/ml IFN-γ or various concentrations of LPS alone overnight. Brefeldin A was added to the culture for 5 h before intracellular staining. Cells were fixed with IC Fixation Buffer (eBioscience), incubated with permeabilization buffer and stained with PE-conjugated anti-mouse IL-12p40, IL-6, TNF, IL-1β, IL-10, APC-conjugated anti-iNOS, FITC-conjugated anti-mouse CD11b and Alexa Fluor 700-conjugated anti-mouse CD11c antibodies. Samples were analyzed on an LSR Fortessa analyzer (BD Biosciences).

### RNA isolation and quantitative real-time RT–PCR (qPCR)

Total RNA was extracted using an RNeasy plus kit (QIAGEN), and cDNA was generated with an oligo (dT) primer and the Superscript II system (Thermo Fisher Scientific) followed by analysis using iCycler PCR with SYBR Green PCR master Mix (Applied Biosystems). Program was chosen to compare the C_T_ value of target gene to that of the housekeeping gene Ubiquitin in a single sample, using the formula: 10000×2ΔΔCT. The following primer sets were used: IL-6 sense, 5'-CCAGAAACCGCTATGAAGTTCCT-3', IL-6 anti-sense, 5'-CACCAGCATCAGTCCCAAG-A-3'; IL-12p40 anti-sense, 5'-TCTTCAAAGGCTTCATCTGCAA-3'; TNF sense, 5'- GCC-ACCACGCTCTTCTGTCT-3', TNF anti-sense, 5'-GGTCTGGGCCATAGAACTGATG-3'; Ubiquitin sense, 5'-TGGCTATTAATTATTCGGTCTGCAT-3', Ubiquitin anti-sense, 5'- GCA-AGTGGCTAGAGTGCAGAGTAA-3'; iNOS sense, 5'-CCGAAGCAAACATCACATTCA-3', iNOS anti-sense, 5'-GGTCTAAAGGCTCCGGGCT-3'.

### T cell proliferation assay

CD4^+^ T cells were purified from spleens and lymph nodes of OT-II mice and the cells were labelled with CFSE. The labelled cells (1×10^5^ per well) were co-cultured with dendritic cells in the absence or presence of OVA323-339 peptide for 3 days in 96-well microplates. CFSE dilution was assayed by flow cytometry.

### Immunoblotting analysis

Cells were washed with cold PBS and lysed for 15 min on ice in 0.5ml of lysis buffer (50mM Tris-HCl, pH 8.0, 280mM NaCl, 0.5% Nonidet P-40, 0.2mM EDTA, 2mM EGTA, 10% glycerol and 1mM dithiothreitol) containing protease inhibitors. Cell lysates were clarified by centrifugation (4°C, 15 min, 14,000 r.p.m.) and protein was subjected to 10% sodium dodecyl sulfate–PAGE (SDS–PAGE) and immunoblotting was performed. Anti-iNOS (Santa Cruz), and anti-β-actin (Sigma) antibodies were used according to the manufactures' instructions. Secondary antibodies were from Santa Cruz.

### ChIP assay

BMDCs from WT and iNOS^−/−^ mice were stimulated with IFN-γ (10 ng/ml) and LPS (200 ng/ml) in the presence of SNAP (500 μM) or L-NIL (40 μM) overnight, followed by ChIP assay manuscript. Three micrograms of an anti-NF-kB p65 antibody or isotype-matched IgG as control antibody were used in the immunoprecipitation step. PCR was used to quantify the amount of precipitated DNA with primers flanking the p65-binding site of the IL-12 promoter region.

### Bacterial infection of mice

*Citrobacter rodentium* strain DBS100 (ATCC 51459) was prepared by overnight shaking at 37°C in Luria-Bertani broth. Bacterial cultures were serially diluted and plated on MacConkey agar plates so the CFU dose administered could be confirmed. For infection, C57BL/6 or *iNOS*^−/−^ mice were fasted for 8 h before oral inoculation with 2×10^9^ CFU of *C. Rodentium* in a total volume of 100 μl per mouse. Mortality was monitored daily throughout the infection. Body weights were assessed at the beginning of infection and every 2 days after infection.

### Endotoxin-induced sepsis shock

C57BL/6 or *iNOS*^−/−^ mice were intraperitoneally administered with 800 μg E.coli-derived ultra-pure LPS or PBS. Survival was monitored continuously. Mice were euthanized at a humane end point after loss of self-righting (capability to right itself after falling) and insensitivity to touch were noted. For serum collection, mice were injected i.p. with 800 μg LPS and sera were collected 6 h later. Spleens were collected as well.

### Tissue collection, histology and bacterial load determination

Colons were dissected from mice and fixed with 4% (vol/vol) paraformaldehyde. Paraffin-embedded tissue sections were stained with hematoxylin and eosin for evaluation of tissue pathology. Colons and spleens were removed aseptically, weighed and homogenized in PBS. Homogenates were serially diluted and plated on MacConkey agar plates for determination of bacterial load. *C. rodentium* colonies (pink with white rings) were counted after overnight incubation at 37°C. Fecal specimens were collected, weighed and homogenized in PBS and the CFU counts were determined similarly by plating.

### Statistical analysis

The results are shown as means ± SD. and statistical analysis was performed using Student's t-test. Where more than two groups were compared, one-way ANOVA with a Bonferroni's correction was performed; Student's t-test was used to determine the difference in survival rate. P values <0.05 were considered statistically significant.

## SUPPLEMENTARY FIGURES


