# Supplementary material for: Dendritic cell-derived nitric oxide inhibits the differentiation of effector dendritic cells
Source: Oncotarget. 2016 Aug 18;7(46):74834–45. doi: 10.18632/oncotarget.11361 (PMC5342705; doi:10.18632/oncotarget.11361)
Supplement: Supplementary file 1 [file oncotarget-07-74834-s001.pdf]

Dendritic cell-derived nitric oxide inhibits the differentiation of effector dendritic cells

SUPPLEMENTARY FIGURES

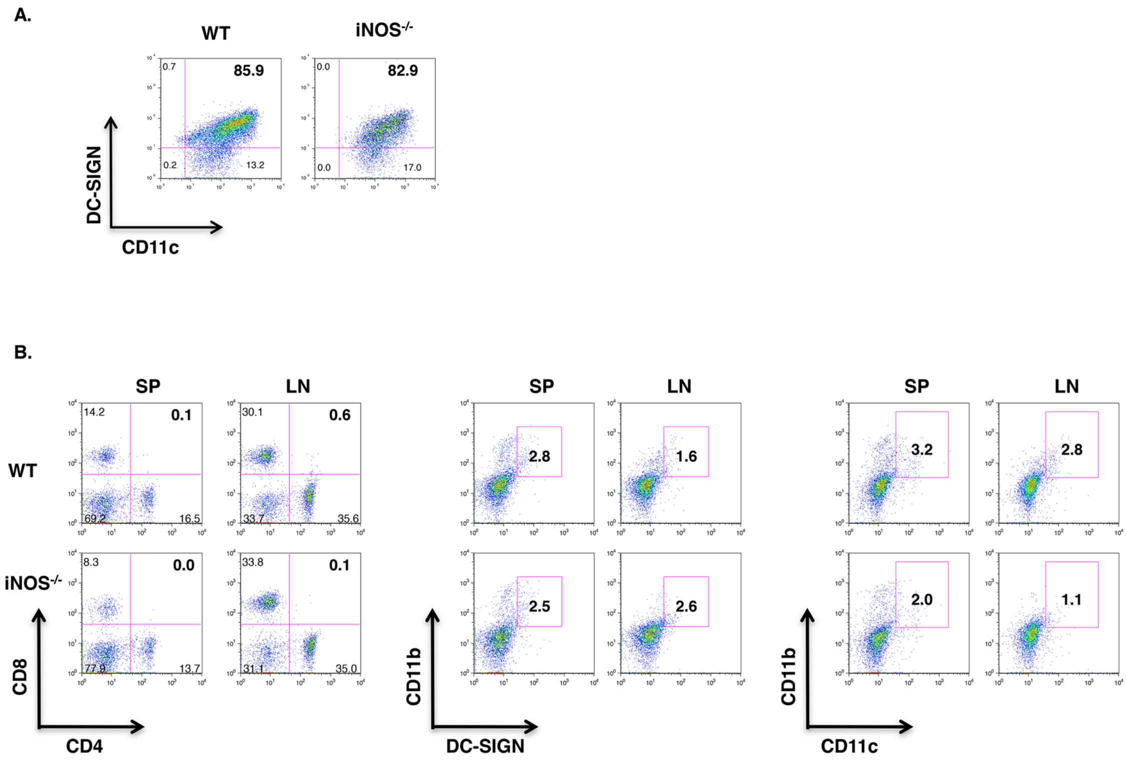

Supplementary Figure S1: T cells and DCs development in iNOS-deficient mice was comparable with that in WT mice. A. Bone marrow cells from WT and iNOS$^{-/-}$ mice were cultured with GM-CSF (10ng/ml) and IL-4 (10ng/ml) for 7 days, and DC cell markers were analyzed by FACS. **B.** CD4^{+} and CD8^{+} T cells population and DCs percentage in spleen and lymph node from WT or iNOS$^{-/-}$ mice were analyzed by FACS.

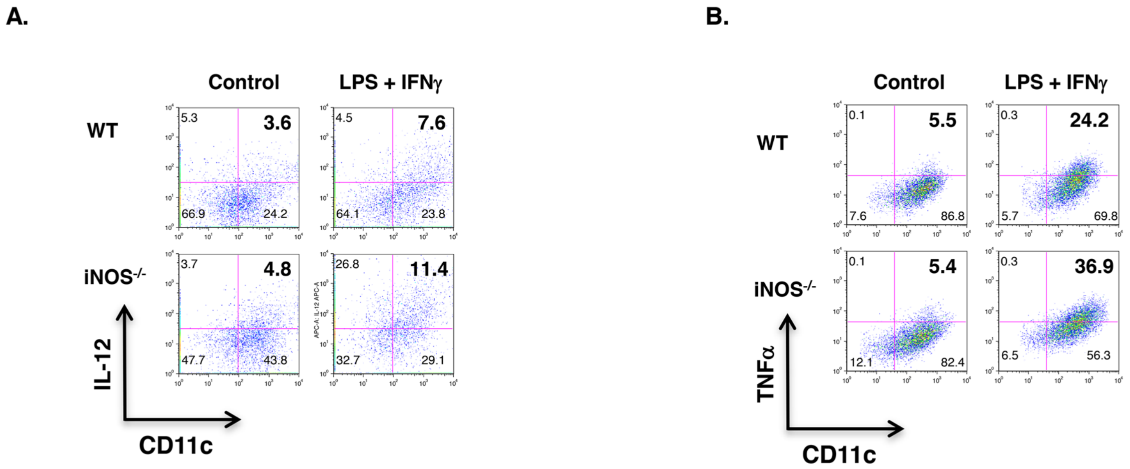

Supplementary Figure S2: NO suppress effective DCs differentiation. Bone Marrow DC cells from WT and iNOS$^{-/-}$ mice were stimulated with IFN-γ plus LPS for overnight, IL-12 **A.** or TNFα **B.** expression in CD11b$^+$CD11c$^+$ DCs were analyzed by FACS.

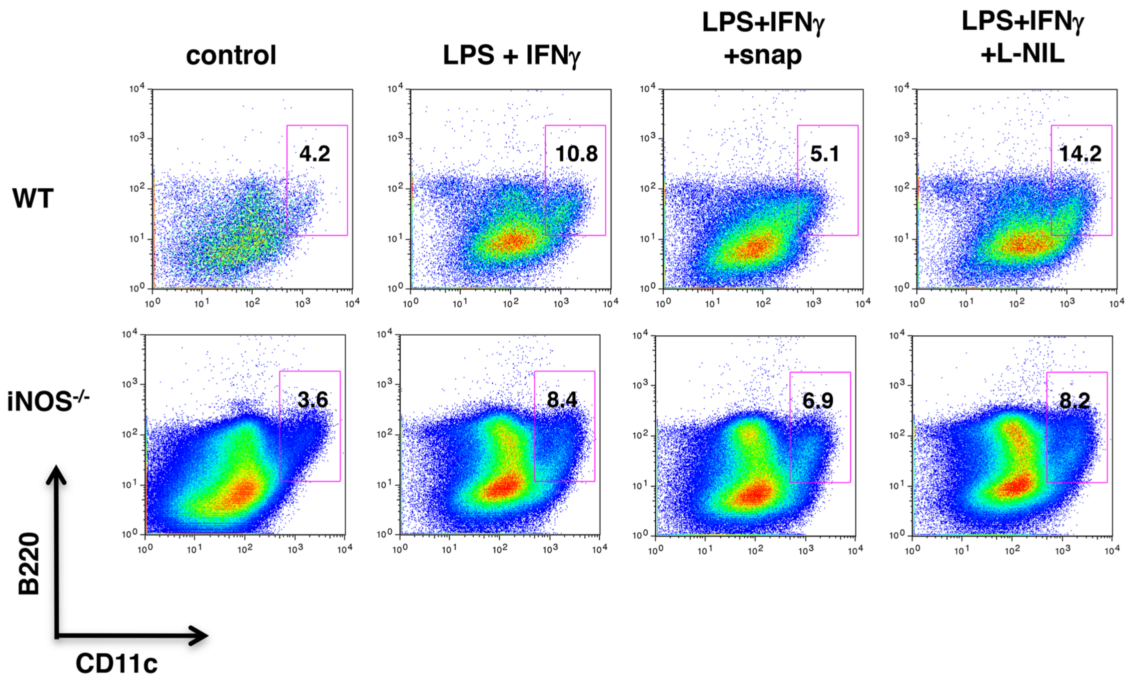

Supplementary Figure S3: NO did not affect pDC differentiation in short time. Bone Marrow cells from WT and iNOS$^{-/-}$ mice were stimulated with IFN-γ plus LPS for overnight in the presence of SNAP or L-NIL, CD11c^{+}B220^{+} pDC percentages were analyzed by FACS.

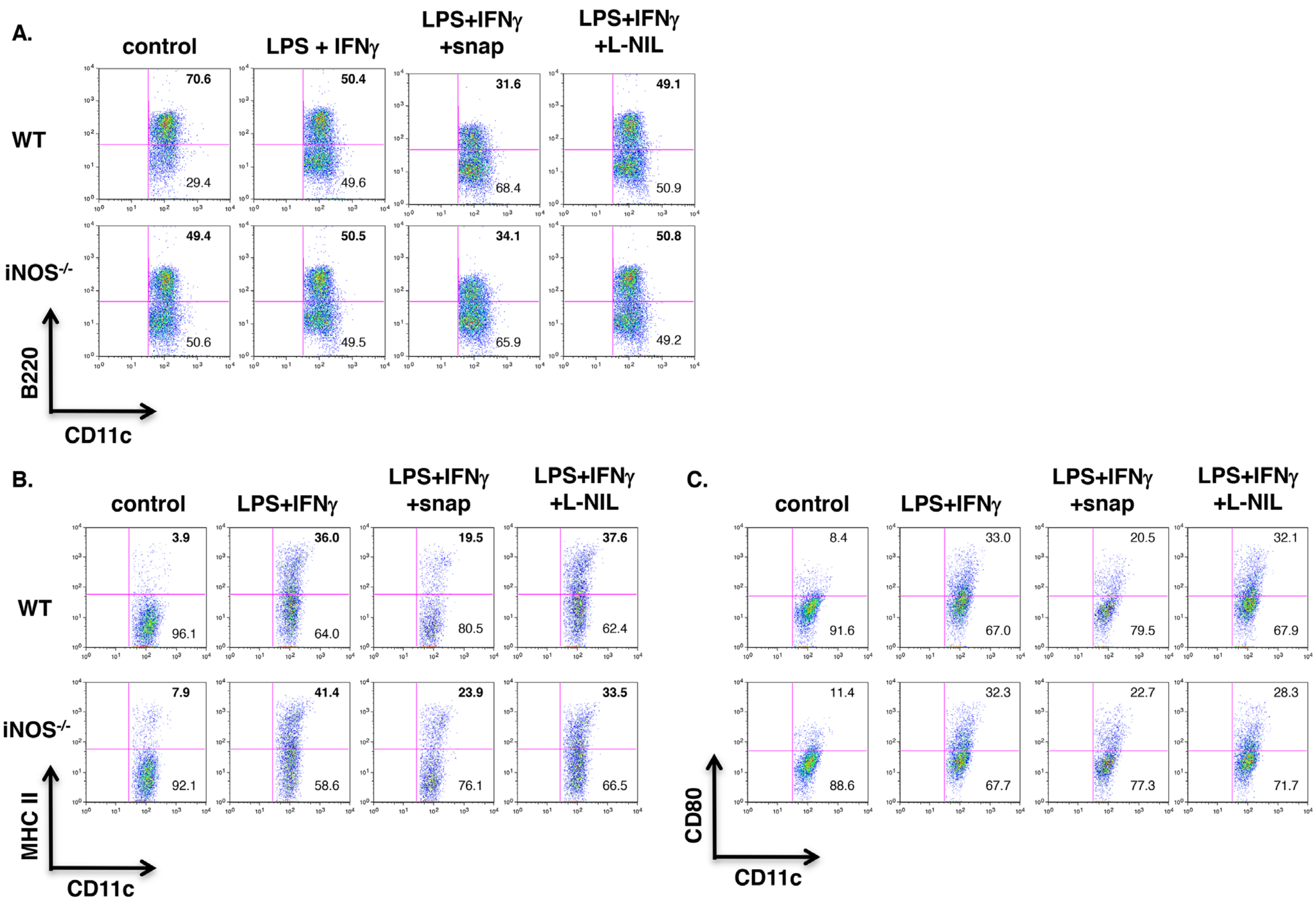

Supplementary Figure S4: NO suppresses maturation and differentiation of pDC *in vitro*. Bone Marrow cells from wild type or iNOS$^{-/-}$ mice were cultured with Flt3-L for 7 days, then stimulated with IFN-γ plus LPS for 24 h, CD11c^{+}B220^{+} pDC percentages **A.** were analyzed by FACS. and maturation markers including MHC-II^{+} **B.** and CD80^{+} pDC **C.** were analyzed by FACS.
